# Role of Galactolipids in Plastid Differentiation Before and After Light Exposure

**DOI:** 10.3390/plants8100357

**Published:** 2019-09-20

**Authors:** Sho Fujii, Hajime Wada, Koichi Kobayashi

**Affiliations:** 1 Department of Botany, Graduate School of Science, Kyoto University, Oiwake-cho, Kita-Shirakawa, Sakyo-ku, Kyoto 606-8502, Japan; shofujii@pmg.bot.kyoto-u.ac.jp; 2 Department of Life Sciences, Graduate School of Arts and Sciences, The University of Tokyo, 3-8-1 Komaba, Meguro-ku, Tokyo 153-8902, Japan; hwada@bio.c.u-tokyo.ac.jp; 3 Faculty of Liberal Arts and Sciences, Osaka Prefecture University, 1-1 Gakuen-cho, Naka-ku, Sakai 599-8531, Japan

**Keywords:** chlorophyll, chloroplast, digalactosyldiacylglycerol, etioplast, galactolipid, monogalactosyldiacylglycerol, photosynthetic proteins, prolamellar body, thylakoid membrane

## Abstract

Galactolipids, monogalactosyldiacylglycerol (MGDG) and digalactosyldiacylglycerol (DGDG), are the predominant lipid classes in the thylakoid membrane of chloroplasts. These lipids are also major constituents of internal membrane structures called prolamellar bodies (PLBs) and prothylakoids (PTs) in etioplasts, which develop in the cotyledon cells of dark-grown angiosperms. Analysis of Arabidopsis mutants defective in the major galactolipid biosynthesis pathway revealed that MGDG and DGDG are similarly and, in part, differently required for membrane-associated processes such as the organization of PLBs and PTs and the formation of pigment–protein complexes in etioplasts. After light exposure, PLBs and PTs in etioplasts are transformed into the thylakoid membrane, resulting in chloroplast biogenesis. During the etioplast-to-chloroplast differentiation, galactolipids facilitate thylakoid membrane biogenesis from PLBs and PTs and play crucial roles in chlorophyll biosynthesis and accumulation of light-harvesting proteins. These recent findings shed light on the roles of galactolipids as key facilitators of several membrane-associated processes during the development of the internal membrane systems in plant plastids.

## 1. Introduction

Plastids are a diverse family of plant organelles probably derived from a cyanobacterial ancestor through endosymbiosis. In higher plants, various types of plastids differentiate from undifferentiated proplastids or other types of plastids, depending on the host cell type and developmental stages [1]. Chloroplasts are the most typical form of plastids that develop the thylakoid membrane inside to perform oxygenic photosynthesis. Under light, most chloroplasts differentiate directly from proplastids. However, in the absence of light, as often observed in angiosperms germinated in the dark, proplastids in leaves differentiate to precursors of chloroplasts, etioplasts [2].

Etioplasts largely differ from chloroplasts, particularly in structures and components of internal membranes. Unlike chloroplasts with highly stacked lamellar thylakoid membranes, etioplasts form paracrystalline, three-dimensional lattice structures named prolamellar bodies (PLBs), from which flattened lamellar prothylakoids (PTs) are radiated [2]. Etioplasts accumulate protochlorophyllide (Pchlide), the precursor of chlorophyll (Chl), in these internal membranes. With light exposure, etioplasts rapidly differentiate to chloroplasts to establish photoautotrophic growth. The differentiation from etioplasts to chloroplasts after light exposure involves the dynamic transformation of PLBs to thylakoids, which is accompanied by changes in pigment compositions from Pchlide to Chl and accumulation of other photosynthetic components including photosystem (PS) I, PSII, and light-harvesting complex (LHC).

**Figure 1:**
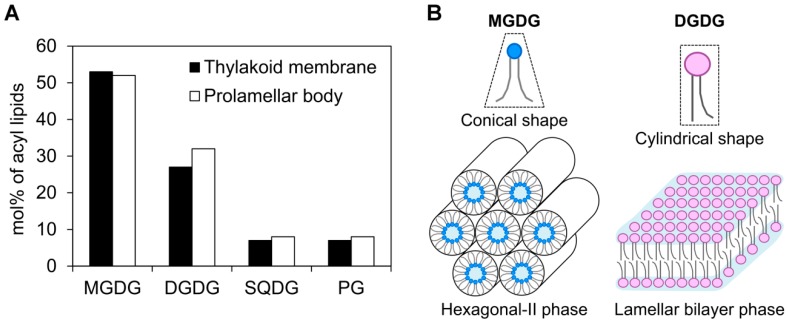
Abundance of galactolipids in plastid internal membranes and their structural characteristics. (**A**) Glycerolipid composition in the thylakoid membrane from spinach chloroplasts [8] and prolamellar bodies from wheat etioplasts [7]. Only the major lipids are compared. (**B**) Conceptual structure of monogalactosyldiacylglycerol (MGDG) and digalactosyldiacylglycerol (DGDG) and their phase behaviors in aqueous mixtures.

The biogenesis of the thylakoid membrane requires coordinated biosynthesis and assembly of photosynthetic membrane proteins, pigments, and cofactors with glycerolipids. Glycerolipids in the thylakoid membrane provide a lipid bilayer matrix for photosynthetic complexes responsible for photochemical and electron transport reactions and allow for generating an electrochemical potential difference across the membrane for ATP synthesis. In addition, glycerolipids function directly in photosynthesis as structural components of PSII, PSI, LHCII, and cytochrome *b*_6_*f* complexes [3]. The lipid matrix of the thylakoid membrane consists mainly of four lipid classes—monogalactosyldiacylglycerol (MGDG), digalactosyldiacylglycerol (DGDG), sulfoquinovosyldiacylglycerol (SQDG), and phosphatidylglycerol (PG) (Figure 1A)—each of which has specific roles in the biogenesis and maintenance of the thylakoid membrane and photosynthesis.

Of the four major thylakoid lipids, galactolipids MGDG and DGDG constitute the bulk of the lipid bilayer and thus play essential roles in chloroplast biogenesis [4]. MGDG has a conical shape with a small head group of a single galactose moiety and flexible poly-unsaturated fatty acid tails and forms a hexagonal-II phase in aqueous mixtures. In contrast, DGDG has a cylindrical shape with a larger head group of two galactose moieties, which allows for forming a lamellar bilayer phase (Figure 1B) [5]. The ratio of non-bilayer-forming MGDG to bilayer-forming DGDG is important to form and stabilize the thylakoid membrane structure [6]. The MGDG-to-DGDG ratio is also suggested to be required for the unique structures of PLBs and PTs in etioplasts [7]. Recently, characterization of galactolipid-deficient Arabidopsis mutants have revealed similar and different involvements of MGDG and DGDG in etioplast development in the dark and etioplast-to-chloroplast differentiation after light exposure.

In this review, we briefly outline internal membrane processes during etioplast development in the dark and etioplast-to-chloroplast differentiation after light exposure and discuss the roles of MGDG and DGDG in these processes. Recent advances in understanding the functions of galactolipids as key facilitators of various membrane-associated processes in etioplasts and chloroplasts provide important insights into the complex processes of thylakoid membrane development and photosynthetic activation in plants.

## 2. Development of Etioplasts in the Dark and Their Differentiation to Chloroplasts with Light

### 2.1. Pchlide and Chl Biosynthesis in the Dark and in the Light

**Figure 2:**
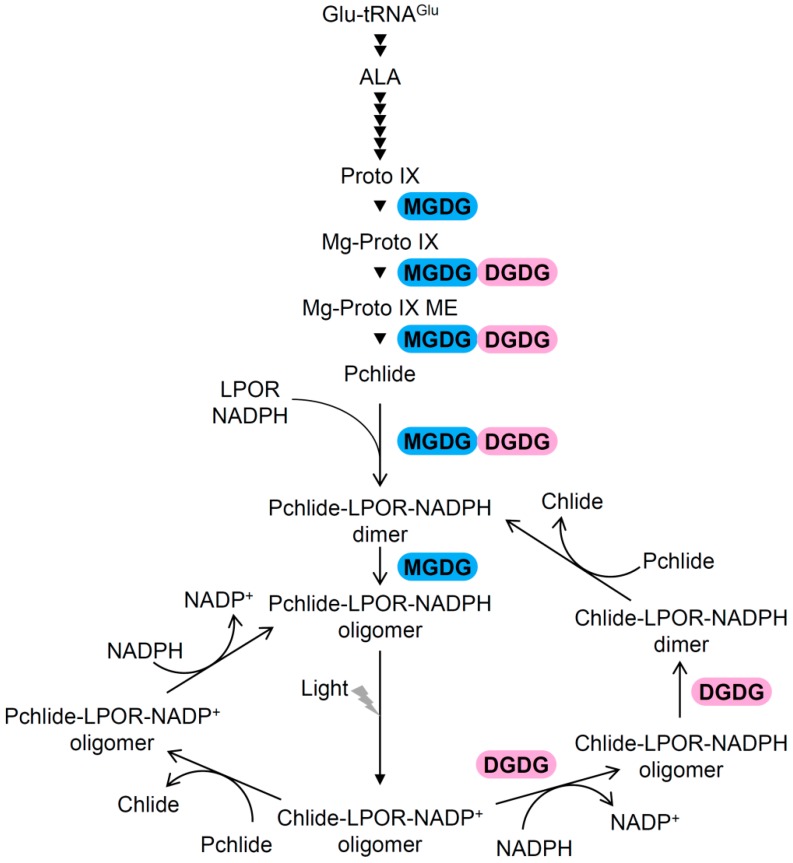
Involvements of galactolipids in Pchlide biosynthesis, the organization of Pchlide–LPOR–NADPH complexes and the regeneration of photoactive ternary complexes after photoconversion. The pathways for Chlide biosynthesis and the conversion of (P)chlide–LPOR complexes are shown with important intermediates. An involvement of MGDG and/or DGDG is indicated by their names with the corresponding steps. Both galactolipids facilitate the membrane-associated processes of the Pchlide biosynthesis pathway and the formation of the Pchlide–LPOR–NADPH ternary complex. In addition, MGDG plays a role in the oligomerization of the ternary complex, whereas DGDG is required for the modification of the complex after photoconversion.

Plants synthesize tetrapyrroles, including Pchlide and Chl, in plastids as described in comprehensive reviews (Figure 2) [9,10,11,12]. As an early step of tetrapyrrole biosynthesis, the generation of 5-aminolevulinic acid (ALA) from glutamyl-tRNA^Glu^ is a rate-limiting step of the entire pathway. ALA is processed into protoporphyrin IX (Proto IX), the last common intermediate for both Chl and heme biosynthesis, by a cascade of several enzymatic steps. For Chl synthesis, Mg-chelatase (MgCh) inserts Mg^2+^ into Proto IX to yield Mg-Proto IX, followed by esterification of Mg-Proto IX to Mg-Proto IX monomethylester (Mg-Proto IX ME) by S-adenosyl-l-methionine:Mg-Proto IX methyltransferase (MgMT). Subsequently, Mg-Proto IX ME cyclase (MgCY) introduces the fifth ring to Mg-Proto IX ME to form divinyl Pchlide, which is then reduced to chlorophyllide (Chlide) by Pchlide reductase (POR). After reduction of the 8-vinyl group, Chlide *a* is esterified with geranylgeraniol or phytol by Chl synthase to form Chl *a*. Some of Chl *a* is reversibly converted to Chl *b* via the Chl cycle.

Most oxygenic photosynthetic organisms can synthesize Chl both in the light and in the dark. Exceptions include angiosperms, which synthesize Chl exclusively in a light-dependent manner. In angiosperms, the conversion of Pchlide to Chlide is catalyzed by light-dependent NADPH:Pchlide oxidoreductase (LPOR), which absolutely requires light for its enzymatic activity [13]. Unlike angiosperms, other oxygenic phototrophs including cyanobacteria can convert Pchlide to Chlide in the absence of light, because these organisms have another type of POR active in the dark, the so-called dark-operative POR, in addition to LPOR [14]. The light dependence of the Pchlide reduction in angiosperms led to the establishment of a specific light-responsive developmental program called greening or deetiolation.

### 2.2. Formation of Pchlide–LPOR–NADPH Ternary Complexes in PLBs

Reflecting the light dependent property of LPOR to reduce Pchlide, angiosperms accumulate Pchlide with LPOR in the dark. In etioplasts, LPOR binds Pchlide and NADPH to form photoactive ternary complexes. Therefore, the abundance of LPOR proteins affects the formation of the photoactive complex [13,15]. In addition, carotenoids are required for accumulation of the photoactive complex [16,17], probably without largely affecting LPOR protein levels [18]. Because xanthophylls are strongly associated with LPOR in wheat etioplasts [19], these pigments may stabilize the Pchlide–LPOR–NADPH complex on the membrane. In etioplast membranes, the photoactive ternary complex further forms dimeric or large oligomeric complexes (Figure 2) [20]. The oligomeric complex is the major form especially in PLBs, whereas small amounts of the dimeric complex are identified in both PLBs and PTs, with relatively enriched in PTs in wheat etioplasts [21,22].

With illumination, LPOR in the photoactive complex instantaneously reduces Pchlide to Chlide by using NADPH as a reductant [23]. Although Pchlide is a photosensitizer with a potential to generate singlet oxygen under light, the formation of the photoactive complex prevents photooxidative damage from light-absorbed Pchlide [2]. After the photoconversion of Pchlide, the resulting Chlide–LPOR–NADP^+^ oligomeric complex is processed in two different pathways to regenerate the photoactive Pchlide–LPOR–NADPH complex (Figure 2) [20]. In the major pathway, NADP^+^ is replaced by NADPH to form the Chlide–LPOR–NADPH ternary complex, which contributes to prevent photodamage from Chlide [2]. This process is followed by dissociation of the oligomeric complex to dimeric complexes and replacement of Chlide by Pchlide. In the minor pathway, the replacement of the pigments and cofactors occurs in the oligomeric complex, which would be advantageous to regenerate the photoactive complex rapidly [24]. Meanwhile, some portion of Pchlide, referred to as nonphotoactive Pchlide, is not bound to the active site of LPOR and thus is inconvertible by a short illumination [14,25]. Reflecting the photosensitizing nature of Pchlide, an excess accumulation of nonphotoactive Pchlide often causes photobleaching of seedlings with light [26].

### 2.3. Formation of PLBs in Etioplasts

**Figure 3:**
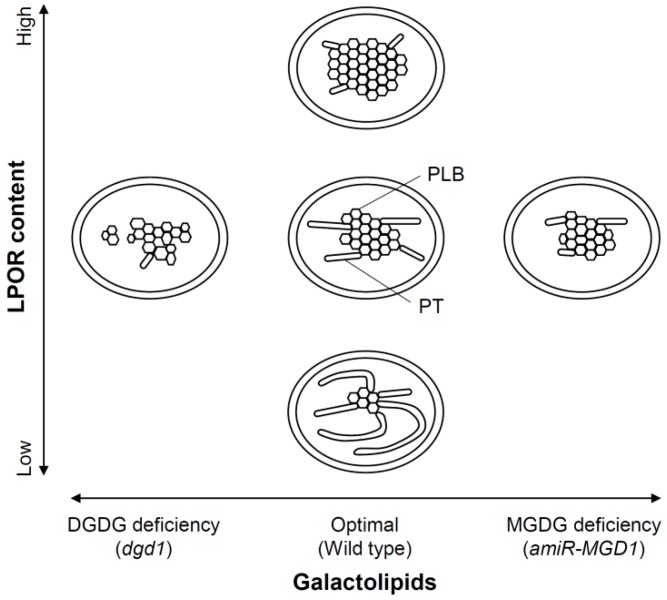
Role of galactolipids and NADPH:Pchlide oxidoreductase (LPOR) proteins in the formation of etioplast membranes. The size of PLBs correlates with the amount of LPOR proteins whereas the structure of PLBs depends on the galactolipid composition. Impaired galactolipid biosynthesis reduces the size of PTs, presumably via decreasing total lipid content and/or changing the lipid composition.

In etioplasts, LPOR is the most abundant protein and particularly enriched in PLBs [7,27], whereas most proteins in the photosynthetic machinery including PSI, PSII, and LHCs are very minor and, if present, are not assembled into mature complexes [28]. In Arabidopsis, there are three isoform genes (*PORA*, *PORB*, and *PORC*) for the LPOR activity. PORA and PORB are highly expressed in the dark and accumulated in etioplasts as major LPOR isoforms, whereas PORC is light-inducible and mainly functions under light conditions [13]. The levels of PORA and PORB strongly affect the size of PLBs in Arabidopsis. A decrease in the total amount of LPOR proteins by knockout mutations of *PORB* [29,30] or antisense RNA-mediated suppression of *PORA* or *PORB* [15] diminished the size of PLBs in etiolated Arabidopsis seedlings. The loss-of-function mutation in CONSTITUTIVE PHOTOMORPHOGENESIS 1 (COP1), a ubiquitin E3 ligase suppressing photomorphogenesis in the dark, strongly decreased the expression of *PORA* and *PORB* and inhibited the formation of PLBs in dark-grown Arabidopsis [31]. However, constitutive expression of either *PORA* or *PORB* in the *cop1* mutant restored PLBs. Moreover, overaccumulation of either of the LPOR isoforms in etioplasts enlarged the size of PLBs [15,32]. These data suggest that the total amount of LPOR proteins determines the size of PLBs in etioplasts (Figure 3) [13,15].

In contrast to the size of PLBs, the lattice structure of PLBs is independent of the amount of LPOR proteins [15,29,30,32,33,34]. Instead, the biosynthesis of carotenoids is deeply involved in the formation of the regular lattice structure of PLBs. Treatment of amitrole, which inhibits lycopene cyclization, and the knockout mutation of a gene encoding carotenoid isomerase, which is responsible for all-trans-lycopene synthesis, strongly disturbed the structure of PLBs and the formation of photoactive Pchlide, whereas norflurazon treatment, which inhibits phytoene desaturation, did not largely alter the PLB development [16,17]. These results indicate that excess accumulation of ζ-carotene, neurosporene, and/or cis-lycopene, which are almost undetectable in etiolated wild-type seedlings, inhibits the organization of the PLB lattice structure. Carotenoids abundant in etioplasts, such as lutein and violaxanthin [19], are likely important for stabilization of the lattice structure of PLBs [17].

### 2.4. Transformation of PLBs to the Thylakoid Membrane During Etioplast-to-chloroplast Differentiation

Electron tomography observation of dark-grown runner bean seedlings revealed that, after 1 h of illumination, a regular tetrahedral structure of tubular connections within PLBs becomes irregular and porous PTs surround the degrading PLBs radially [35]. The PLB degradation further proceeds and porous PTs are arranged parallel to each other during subsequent hours of illumination. Stacked membranes connected with PTs appear after 8 h of illumination and the size of grana increases during chloroplast maturation. 

With illumination, Pchlide in the photoactive complex is instantaneously converted to Chlide, which is further metabolized to Chl by downstream enzymes [36,37]. In parallel, light signals upregulate expression of genes for Chl biosynthesis and activate de novo Chl biosynthesis [2]. Virtually all Chl molecules are bound to photosynthetic complexes, particularly LHCII [38]. Many mutants deficient in Chl biosynthesis lack LHCII with severely impaired thylakoid biogenesis and grana stacking, which indicates the necessity of Chl for the accumulation of LHCII and thylakoid membrane biogenesis [29,39,40,41]. Chl biosynthesis is also required for the accumulation of D1, the core PSII protein, and assembly of PSII [28].

Proteins occupy 70–80% of the thylakoid membrane surface [42] and thus play essential roles in thylakoid biogenesis during chloroplast development. In Arabidopsis, rapid degradation of PORA and PORB was observed with illumination to etiolated seedlings [43]. Considering that the LPOR protein level is a determinant factor of the PLB size, the degradation of LPOR proteins would induce the dispersion of PLBs. Although some photosynthetic membrane proteins such as subunits of ATP synthase and cytochrome *b*_6_*f* are accumulated in etioplasts [44], the components of major photosynthetic complexes including LHCII, PSI, and PSII are almost absent and not assembled into mature complexes in dark-grown seedlings [28,45]. During the etioplast-to-chloroplast differentiation in pea seedlings, the amount of the core components of PSI, PSII, LHCI, and LHCII rapidly rose in 6 h of illumination and further increased in 24 h, whereas the amount of LPOR proteins gradually decreased [46]. Photosynthetic protein-pigment complexes are assembled in parallel with the transformation from PLBs to the thylakoid membrane [35]. LHCII and PSII are abundant in grana regions of the thylakoid membrane, whereas PSI is enriched in stroma lamellae in mature chloroplasts [47]. Disruption of either PSI or PSII severely perturbs the structure of the thylakoid membrane in chloroplasts, with PSI particularly affecting the formation of stroma lamellae whereas PSII being more important for the grana stacking. LHCII is also essential for the development of the thylakoid membrane and particularly grana formation, as evidenced by disturbances of the grana structure in mutants or transgenic plants in which the accumulation or organization of LHCII is altered [48,49,50,51]. In addition, CURVATURE THYLAKOID1 (CURT1) protein family functions in grana stacking independently of the mechanism mediated by LHCII. CURT1 proteins are localized to the grana margin and probably induce membrane curvature directly [52].

## 3. Role of Galactolipids in Etioplasts

### 3.1. Galactolipid Synthesis in Etioplasts

In addition to proteins and pigments, glycerolipids are essential components of internal membranes of etioplasts and chloroplasts. The composition of glycerolipids is similar between PLBs and the thylakoid membrane (Figure 1A), despite the large differences in their structures. In both PLBs in etioplasts [7] and the thylakoid membrane in chloroplasts [8], MGDG and DGDG account for ~50% and ~30%, respectively, of total membrane lipids. PTs have a relatively lower MGDG-to-DGDG ratio (1.1) than PLBs (1.6) in wheat etioplasts [7].

Plant MGDG is synthesized by MGDG synthase in the plastid envelope, which transfers a galactose moiety from UDP-galactose to diacylglycerol. Arabidopsis possesses three isoforms of MGDG synthase, namely inner-envelope localized MGD1 and outer-membrane localized MGD2 and MGD3 [53]. DGDG synthase in the outer envelope of plastids synthesizes DGDG by transferring a galactose moiety from UDP-galactose to MGDG. Arabidopsis has two isoforms of DGDG synthase, namely DGD1 and DGD2 [54,55]. MGD1 and DGD1 synthesize the bulk of galactolipids in chloroplasts whereas MGD2, MGD3 and DGD2 mainly function to provide DGDG to the extraplastidic membranes specifically under phosphate-starved conditions [56]. In etiolated seedlings, knockout mutations of both *MGD2* and *MGD3* did not alter galactolipid content, which suggests that *MGD2* and *MGD3* are not involved in galactolipid synthesis in etioplasts [57]. A knockout mutant of *MGD1* (*mgd1-2*) fails to develop cotyledons during embryogenesis and is not useful to reveal the *MGD1* function in etiolated seedlings [58]. Meanwhile, a recent analysis of Arabidopsis transgenic lines expressing an artificial microRNA targeting *MGD1* (*amiR-MGD1*) under a dexamethasone-inducible promoter revealed that suppression of the *MGD1* mRNA level to 35% of control plants decreased MGDG content to 64% of the control without affecting DGDG content in etiolated seedlings [59]. These data suggest that MGD1 is the major enzyme responsible for MGDG synthesis in etioplasts. DGDG biosynthesis in etioplasts is mainly catalyzed by DGD1 in Arabidopsis. A knockout mutation of *DGD1* (*dgd1*) repressed DGDG accumulation to 20% of the wild type with only a small decrease in MGDG content [60]. The remaining DGDG in *dgd1* may be synthesized by DGD2.

### 3.2. Involvement of Galactolipids in Pchlide Biosynthesis

Galactolipid deficiency affects the biosynthesis of Pchlide in etiolated Arabidopsis seedlings. In etiolated *MGD1*-suppressed *amiR-MGD1* and *dgd1* seedlings, the amount of Pchlide was decreased to 60% and 76%, respectively, of the control level [59,60]. Treatment of excess amount of ALA, which bypasses the rate-limiting step of the Pchlide biosynthesis pathway, sharply increases the amount of Pchlide in etiolated wild-type seedlings. However, in galactolipid-deficient seedlings, exogenous ALA treatment caused the accumulation of Pchlide intermediates such as Proto IX, Mg-Proto IX, and Mg-Proto IX ME, instead of a strong increase in the Pchlide content. Of these porphyrin intermediates, Mg-Proto IX showed the highest accumulation in both galactolipid-deficient seedlings. These results indicate that the membrane-associated steps of the Pchlide biosynthesis pathway, particularly the Mg-Proto IX metabolism by MgMT, is sensitive to the membrane lipid environment in etioplasts. Despite the milder loss of galactolipids in *amiR-MGD1* (36% decrease of MGDG) than *dgd1* (80% decrease of DGDG), *amiR-MGD1* seedlings showed a more severe impairment of Pchlide biosynthesis, suggesting a particular importance of MGDG in Pchlide synthesis (Figure 2).

Although MGDG and DGDG are required for the porphyrin metabolism by MgCh and MgMT in vivo [59,60], both lipids are not essential cofactors of recombinant MgCh and MgMT proteins [61,62,63]. Moreover, Chen et al. [64] recently reported that recombinant *Synechocystis* proteins for MgCh, MgMT, MgCY, LPOR, divinyl reductase, Chl synthase, and geranylgeranyl reductase can generate Chl from intrinsic Proto IX in *E. coli* cells, where galactolipids are completely absent. These Chl synthesizing enzymes are suggested to form heterocomplexes on the plastid membrane for efficient channeling of the intermediates [10,12], so the existence of galactolipids and/or a proper MGDG-to-DGDG ratio may be required for the sublocalization of proteins such as MgCh, MgMT, and MgCY or the complex formation of these proteins. We cannot exclude the possibility that galactolipids enhance the activity of the membrane-bound enzymes in vivo.

Carotenoid biosynthesis also affects the Pchlide biosynthesis in etioplasts. Inhibition of lycopene cyclization in barley enhances the accumulation of ALA, Proto IX, Mg-Proto IX, Mg-Proto IX ME, and even Pchlide, with particularly affecting the steps catalyzed by MgMT and MgCY [18]. Carotenoids may be required for tight regulation of ALA synthesis in the dark and the functionality of membrane-attached MgMT and MgCY. A proper membrane structure built with galactolipids and carotenoids may be essential for the membrane-associated process of the Pchlide biosynthesis pathway and the regulation of ALA biosynthesis, although the effects of galactolipids on ALA synthesis remain unclear.

### 3.3. Roles of Galactolipids in the Organization of (P)chlide–LPOR Complexes Before and After Illumination

In addition to decreased Pchlide content, impaired galactolipid biosynthesis decreases the accumulation of photoactive Pchlide in etiolated Arabidopsis seedlings without largely affecting carotenoid composition, indicating that both MGDG and DGDG are also required for the formation of the ternary complexes (Figure 2) [59,60]. The *MGD1*-suppressed seedlings showed a more severe impairment of photoactive Pchlide accumulation than *dgd1*, which indicates that MGDG is more important than DGDG for the formation of the Pchlide–LPOR–NADPH complexes. Moreover, MGDG deficiency resulted in abnormal accumulation of the dimeric Pchlide–LPOR–NADPH complex [59], whereas the impairment of DGDG or carotenoid biosynthesis did not cause the increase in dimeric complexes [16,17,18,60], so MGDG has a specific role in oligomerization of the ternary complexes (Figure 2). Meanwhile, a loss of DGDG strongly affects the behavior of Chlide–LPOR complexes after photoconversion of Pchlide. Dissociation of oligomeric Chlide–LPOR complexes to dimeric complexes after photoconversion was retarded in *dgd1* compared to the wild type [60], whereas such defect was not observed in *MGD1*-suppressed seedlings [59]. The data indicate that DGDG mediates regeneration of the Pchlide–LPOR–NADPH complex from the Chlide–LPOR–NADP^+^ complex after photoconversion (Figure 2), which would help safe and efficient conversion of Pchlide to Chl during greening of etiolated seedlings.

An in vitro experiment with a mixture of recombinant LPOR proteins and Pchlide isolated from etiolated wheat seedlings revealed the function of thylakoid membrane lipids in the organization of the Pchlide–LPOR–NADPH complexes [65]. Although the addition of NADPH to the mixture of LPOR and Pchlide in the absence of lipids can trigger the formation of the ternary complexes, the presence of thylakoid anionic lipids SQDG and PG drastically enhanced the rate of the complex formation, with galactolipids showing smaller effects. In agreement with the analysis in galactolipid-deficient Arabidopsis seedlings, addition of MGDG caused a red shift of fluorescence emitted from the Pchlide–LPOR–NADPH complexes, indicative of the oligomerization of the ternary complexes, whereas other lipid classes did not show this effect. Altogether, these data represent the importance of thylakoid lipids and lipophilic pigments for the formation and organization of photoactive Pchlide–LPOR complexes formed in etiolated seedlings. MGDG and DGDG have each specific function in the oligomerization of the ternary complexes and the dissociation of Chlide–LPOR complexes, respectively. Notably, a 36% decrease in MGDG by *MGD1* suppression has strong effects on the formation of photoactive complexes and their oligomerization, without strongly disturbing the PLB structure, representing the specific and essential role of MGDG in the organization of the ternary complexes.

### 3.4. Importance of Galactolipids in the Formation of PLBs and PTs

Besides Pchlide, LPOR and carotenoids, galactolipids have been considered as key players in the organization of PLBs and PTs for a long time. Biochemical analysis of isolated PLBs and PTs from wheat etioplasts revealed a higher MGDG-to-DGDG ratio and a lipid-to-protein ratio in PLBs than PTs [7]. In vitro assay with mixtures of lipids and water found that the PLB-like cubic structure appears when MGDG and DGDG are mixed in a ratio of 1.2:1 or 2:1 [66]. These observations imply that enrichment of non-bilayer-forming MGDG is important for the PLB formation whereas higher accumulation of bilayer-forming DGDG helps the development of lamellar PTs. Interaction between LPOR and membrane lipids, especially MGDG, is also suggested to be important for stabilizing the PLB structure [2,67]. Although a knockdown mutant of *MGD1* in Arabidopsis (*mgd1-1*), which has a T-DNA insertion in the promoter region of *MGD1*, showed no obvious changes in membrane structures of etioplasts as compared with wild-type [68], strong *MGD1* suppression by *amiR-MGD1* decreased the MGDG-to-DGDG ratio from 1.37 to 0.96 and affected the membrane structure of PLBs [59]. As suggested previously [7,66], a high MGDG-to-DGDG ratio may be required for the regular lattice structure of PLB. The development of PTs was also impaired by *MGD1* suppression in *amiR-MGD1*, which suggests that MGDG also has some roles in PT development. The non-bilayer property of MGDG may be important for the PT development. Another possibility is that the decrease in total lipid content by the reduced MGDG synthesis directly leads to reduced PT elongation in the *MGD1*-suppressed etioplasts. Considering that the PLB size was not decreased by *MGD1* suppression [59], a substantial portion of lipid constituents in *MGD1*-suppressed etioplasts may be used for the PLB formation, resulting in decreased PT development. The importance of DGDG for internal membrane formation in etioplasts was revealed by the analysis of the *dgd1* mutant [60]. The lattice structure and the entire shape of PLBs, but not the PLB size, were strongly perturbed in *dgd1* etioplasts. In addition, PT development was severely impaired in *dgd1*, consistent with the hypothesis that bilayer-forming DGDG is important for the PT formation [7]. The stronger effect of DGDG deficiency in *dgd1* than MGDG deficiency in *MGD1*-suppressed *amiR-MGD1* may reflect a more crucial role of DGDG than MGDG in the formation and the organization of the etioplast membranes. However, it should be noted that *dgd1* lost more total galactolipid content than the *MGD1*-suppressed seedlings. In *dgd1*, an overall deficiency of lipid components in the lipid matrix might result in severe disturbances in membrane structures.

### 3.5. A Model for Galactolipid-mediated Etioplast Development

**Figure 4:**
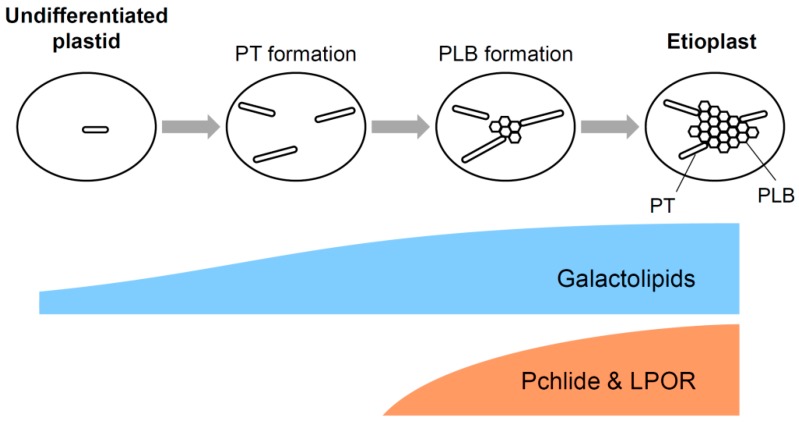
Schematic diagram for a model of galactolipid-mediated etioplast development. Galactolipids accumulated in immature developing etioplasts form lamellar PTs together with anionic lipids. Subsequently, LPOR proteins accumulate with Pchlide on the lipid bilayer and induce the formation of paracrystalline tubular networks with lipids, resulted in the development of prolamellar bodies (PLBs) and maturation of etioplasts.

Considering that galactolipids are required for the Pchlide biosynthesis and the organization of Pchlide–LPOR–NADPH complexes during etioplast development, angiosperms are likely to synthesize and accumulate galactolipids in etioplasts prior to the accumulation of Pchlide and LPOR (Figure 4). At the beginning of etioplast development from undifferentiated plastids, galactolipids may form PT-like lamellar membrane structures because of the lack of LPOR and Pchlide. After initial accumulation of galactolipids in immature etioplast membranes, Pchlide and LPOR may accumulate in the membranes to develop PLBs with lipids. During this process, a proper MGDG-to-DGDG ratio is required to form the highly regular lattice structure of PLBs (Figure 3). Whereas the size of PLBs mainly depends on the LPOR protein levels, the development of lamellar PTs would be strongly affected by the amount and/or composition of lipid constituents. In particular, the amount of DGDG, the main bilayer-forming lipid in etioplasts, may be crucial for the development of PTs, because another major lipid MGDG has a non-bilayer-forming property and is considered to require specific proteins such as LHCII, which is almost absent in etioplasts, to form lamellar membranes. Molecular mechanisms for coordinating biosynthesis of galactolipids, Pchlide and LPOR and the formation of the photoactive ternary complex during etioplast development remain to be elucidated in future studies.

## 4. Role of Galactolipids During Etioplast-to-chloroplast Differentiation

### 4.1. Contribution of Galactolipids to Transformation of PLBs to the Thylakoid Membrane

Upon light exposure, tubular PLBs were directly converted to the lamellar thylakoid membrane [35]. The regular structure of PLBs is rapidly lost in 1–4 h of illumination and transformed into flat slats. Subsequently, stacked membranes appear in the first day of illumination, followed by the development of the grana stacks connected to unstacked stroma lamellae in the next few days. Despite the rapid and dynamic transformation of the internal membrane systems during the etioplast-to-chloroplast differentiation, galactolipid content only gradually increases during the greening process. The content of both MGDG and DGDG was almost unchanged during the first 6 h of illumination in Arabidopsis [43] and cucumber [69]. Etiolated barley seedlings even showed a decrease in galactolipid content at the beginning of greening [70]. Then the barley seedlings increased the lipid content after 6 h, which coincided with the grana formation. In etiolated seedlings of cucumber and Arabidopsis, the galactolipid content increased to ~4 fold and 2–3 fold, respectively, after 24 h of illumination [43,69]. Therefore, in these plants, PLB is transformed into the early thylakoid membrane without an increase in galactolipid content, presumably by diverting lipid constituents from PLBs to thylakoid membranes directly.

**Figure 5:**
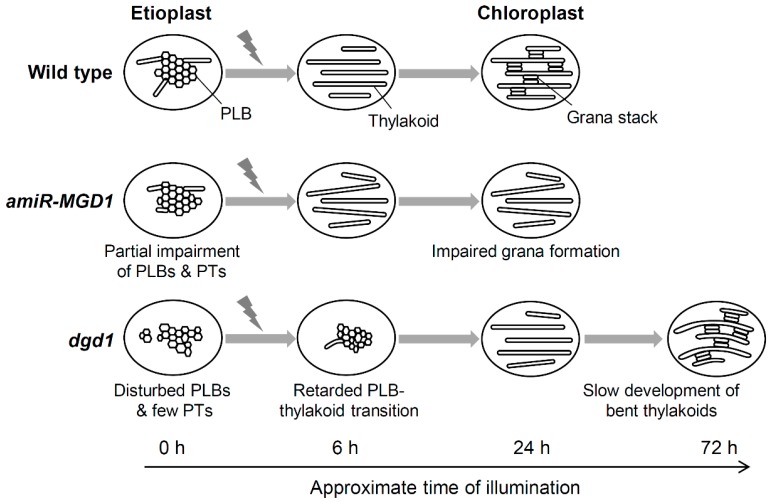
Schematic view of etioplast-to-chloroplast differentiation in galactolipid deficient Arabidopsis seedlings. MGDG deficiency in *MGD1*-suppressed *amiR-MGD1* does not affect PLB-to-lamellar membrane transformation but impairs further membrane development and grana formation during greening. By contrast, DGDG deficiency in *dgd1* seedlings retards PLB dispersion and subsequent lamellar membrane formation at the early stage of greening. However, prolonged illumination gradually induces the development of the highly-stacked thylakoid membrane in *dgd1* chloroplasts, but with an abnormally bent structure.

In *MGD1*-suppressed *amiR-MGD1* and *dgd1* seedlings, both MGDG and DGDG content remained at low levels throughout the greening process [43]. However, in the *MGD1*-suppressed seedlings, PLB was entirely transformed into flat membranes within 6 h of illumination as in control plants, although further development of the thylakoid membrane networks was severely abolished (Figure 5) [43]. Thus, MGDG biosynthesis is unnecessary for the transformation of PLBs to flat membranes at the beginning of greening, but is required for the massive development of thylakoid membranes afterwards. Because the *MGD1*-suppressed seedlings develop PLBs to a size similar to that in control plants, lipids accumulated in the PLBs can directly make flat membranes after PLB dispersion presumably owing to the bilayer-forming nature of DGDG and anionic lipids. Meanwhile, DGDG deficiency by the *dgd1* mutation strongly retarded the PLB-to-thylakoid transition. The remnants of PLBs were still observed after 10 h of illumination in most *dgd1* plastids and even after 24 h in some plastids (Figure 5) [43]. In parallel, the formation of lamellar membranes was strongly impaired in *dgd1* plastids and thylakoid development was severely retarded at least until 24 h of illumination. Remnants of the PLB-like structure were also observed in DGDG-deficient barley seedlings illuminated for 24 h after 7-d dark incubation [71]. Of note, LPOR proteins, which determine the size of PLBs, were rapidly degraded in the *dgd1* seedlings as in the wild-type plants [43]. Thus, the PLB remnants in *dgd1* are not caused by LPOR. In *dgd1* etioplasts after illumination, PLBs rich in MGDG cannot be converted into lamellar membranes even after degradation of LPOR and long remain as membranous aggregates presumably due to hexagonal-phase-forming property of MGDG. These findings underscore an irreplaceable role of DGDG in the initiation of lamellar membrane construction from PLBs during cotyledon greening.

### 4.2. Contribution of Galactolipids to the Development of the Thylakoid Membrane during Chloroplast Maturation

The ratio of non-bilayer-forming MGDG to bilayer-forming DGDG determines the phase behavior of membranes and is likely important for the thylakoid organization [6]. The conical structure of MGDG with a small galactose head group has long been assumed to stabilize the inner leaflet of highly curved grana margins [72]. Recently, Seiwert et al. [73] demonstrated that MGDG promotes membrane stacking by easing the membrane curvature stress in the margin domains. Consistent with these studies, the MGDG-to-DGDG ratio is higher in the grana region than in the stroma lamellae [74], implying the importance of MGDG for grana formation. In fact, knockdown of *MGD1* decreased the development of grana stacks in Arabidopsis [68,75] and tobacco [76], whereas a knockout mutation of *MGD1* (*mgd1-2*) totally inhibits thylakoid biogenesis in Arabidopsis [58]. Moreover, detailed structural analysis of the thylakoid membrane in the heterozygous *mgd1-2* mutant chloroplasts revealed a crucial role of MGDG in the formation of the typical helical grana arrangement and the granum marginal region [77]. In vitro assay and computer simulations demonstrated that DGDG also contributes to grana stacking through an interaction between the head groups [6,78]. However, DGDG deficiency in Arabidopsis *dgd1* and *dgd1 dgd2* mutants even slightly increases the size and numbers of grana stacks under light conditions [79,80]. These seedlings accumulate LHCII to the wild-type level, which probably promotes the formation of grana stacks in the absence of DGDG. Nevertheless, the structure of the entire thylakoid is abnormally bent under light conditions in DGDG-deficient seedlings [77,79,80], highlighting the importance of DGDG in membrane flattening.

As the thylakoid membrane has a high protein density [42], the association of photosynthetic complexes with membrane lipids strongly affects thylakoid membrane structures. In vitro analysis revealed that the interaction of LHCII to non-bilayer-forming MGDG enables MGDG to form a bilayer structure [81]. The data suggest a crucial role of LHCII in the organization of lamellar membranes under an MGDG-rich lipid environment such as grana regions. Meanwhile, MGDG can increase the stability of LHCII against mechanical unfolding in vitro [82], implying a requirement of MGDG for stable accumulation of LHCII in the thylakoid membrane. Furthermore, MGDG and DGDG induce the aggregation of LHCII [83], which may affect thylakoid membrane architecture including grana stacks. As another mechanism, CURT1 proteins, small polypeptides with two transmembrane regions and a tentative N-terminal amphipathic helix, accumulate in grana margins and directly induce membrane curvature [52], although the relationship between CURT1 and membrane lipids is unknown. As described in the Section 2.4., the formation of PSI and PSII in the lipid bilayer would also contribute to the organization of the thylakoid membrane. Therefore, lipids and proteins together make up the thylakoid membrane in a coordinated and interactive manner.

Biosynthesis of Chl is also essential for the thylakoid membrane development, as represented by the impaired thylakoid formation in Chl-deficient mutants [29,39,40,41,49]. Light exposure to etiolated seedlings acutely induces expression of genes for Chl biosynthesis and photosynthesis and triggers the biosynthesis of Chl and thylakoid proteins to assemble photosynthetic protein-pigment complexes. In wild-type Arabidopsis seedlings under the greening process, Chl content increases sharply in response to illumination, whereas LHCII proteins are below detectable levels until 6 h of illumination and drastically accumulated afterwards [43]. In *MGD1*-suppressed *amiR-MGD1* and *dgd1* seedlings, accumulation of Chl was strongly inhibited, probably owing to the same mechanism for the repression of Pchlide synthesis in etioplasts. Accumulation of LHCII proteins was also repressed at low levels in these seedlings at least until 24 h of illumination. Several causes can be considered for the impaired LHCII accumulation in the galactolipid-deficient seedlings. In the *MGD1*-suppressed seedlings, mRNA accumulation of *LHCB1* and *LHCB6* genes was strongly suppressed after an initial increase in response to light [43]. An impaired protein import [84] and destabilization of LHCII [82] by galactolipid deficiency may also cause the decreased LHCII accumulation. In addition, because Chl is necessary for stable accumulation of LHCII, the strong inhibition of Chl biosynthesis would greatly affect accumulation of LHCII and other Chl-binding proteins in the thylakoid membrane. As a result, in addition to galactolipid deficiency, lack of LHCII proteins and other photosynthetic proteins may cause the impaired development of the thylakoid membrane in galactolipid-deficient seedlings. Of note, in the *dgd1* mutant under the greening process, although thylakoid development was severely retarded even at 24 h of illumination, prolonged illumination for 72 h recovered the retardation and resulted in well-developed thylakoid membranes with grana stacks similar to those in the wild type (Figure 5) [43]. These findings agree with the observation that adult *dgd1* plants grown in the light almost fully developed the thylakoid membrane with substantial LHCII accumulation as did wild-type plants [79]. From these data, we hypothesize that, in *dgd1* seedlings under the greening process, slow but gradual accumulation of LHCII and other photosynthetic proteins with Chl enables MGDG-rich lipid constituents to form lamellar membranes and their further development to the thylakoid membrane with grana stacks. By contrast, in *MGD1*-suppressed *amiR-MGD1*, the inhibited Chl accumulation was not recovered by prolonged illumination. Because *MGD1* suppression also inhibited DGDG biosynthesis from MGDG, deficiency of both galactolipids would impair chloroplast development more severely than the single DGDG deficiency. Alternatively, MGDG may have a distinct role in the regulation of chloroplast development and its deficiency may specifically inhibit such processes. In fact, we observed that *MGD1* suppression, but not the *dgd1* mutation, significantly downregulate mRNA expression of nuclear- and plastid-encoded photosynthesis-associated genes [43,85], the mechanism of which should be elucidated in future studies.

### 4.3. A Model for the PLB-to-thylakoid Transformation during Eitoplast-to-chloroplast Differentiation

**Figure 6:**
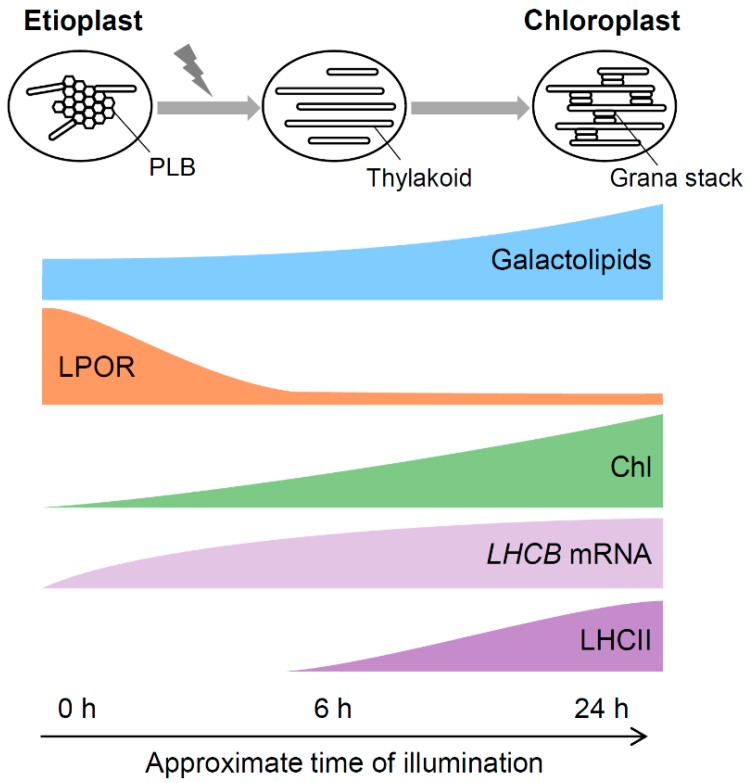
Schematic diagram for a model of etioplast-to-chloroplast differentiation. Illumination to etioplasts induces degradation of LPORs after photoconversion of Pchlide to Chlide, which results in disorganization of the paracrystalline PLB structure. The disorganized PLBs are directly transformed to flat membranes without requiring increases in galactolipid content. In parallel, light activates Chl biosynthesis and mRNA accumulation of *LHCB* genes, which, with a slight delay, induce accumulation of LHCII in developing membranes. Interaction between LHCII and membrane lipids, particularly MGDG, induces multiple stacking of flat membranes, resulting in the development of the thylakoid membrane with grana stacks. At later greening stages, gradually-accumulated galactolipids further extend the thylakoid membrane networks together with photosynthetic proteins to complete chloroplast development and fully activate photosynthesis.

When etiolated seedlings are exposed to light, Pchlide in the photoactive complex is immediately converted to Chlide, and then to Chl. LPOR released from the complex is degraded and the paracrystalline structure of PLBs is disordered (Figure 6). The tubular PLBs were directly converted to the lamellar thylakoid membrane, presumably with the help of bilayer-forming DGDG. In parallel, light signals induce expression of genes for Chl biosynthesis and photosynthesis and activate de novo Chl biosynthesis and photosynthetic protein accumulation. Galactolipid biosynthesis pathways are also activated and galactolipid content gradually increases toward the maturation of the thylakoid membrane. The photosynthetic complexes including LHCII are embedded in lipid bilayer membranes that are transformed from PLBs and PTs or newly synthesized, which enhances the development of thylakoid membrane networks with grana stacking. In this process, non-bilayer-forming MGDG may play a specific role in the formation of the unique thylakoid membrane architecture with highly curved grana margins in collaboration with several membrane proteins. Changes in lipid-protein-pigment interactions result in the dynamic transformation from etioplasts to chloroplasts and the establishment of photoautotrophic growth.

## 5. Perspectives

Together with biochemical and spectroscopic studies, recent studies using genetic and molecular biology techniques have led to uncover the functions of galactolipid biosynthesis in dark-developed etioplasts and their differentiation to chloroplasts. These studies provide new insights into the roles of lipids in organizing several membrane-associated processes during plastid differentiation. However, molecular mechanisms for regulating Chl biosynthesis and photosynthetic gene expression in response to galactolipid synthesis largely remain to be elucidated. Various processes involved in chloroplast development, including lipid and pigment biosynthesis, import of nuclear-encoded proteins and expression of plastid-encoded genes, would take place in, on, or near the envelope membrane at the initial stage of chloroplast development. Considering that galactolipid biosynthesis is a prerequisite for other processes required for chloroplast development [43,75], it may provide the lipid environment that facilitates biosynthesis of pigments and other cofactors, accumulation of proteins from the outside and the inside of plastids, and assembly of photosynthetic complexes. In this sense, galactolipids may act as an initiator of thylakoid membrane biogenesis and thus chloroplast development. Of note, etioplasts and chloroplasts also contain anionic lipids SQDG and PG as major lipid constituents, and their roles in etioplast development and etioplast-to-chloroplast differentiation are also important questions to be answered in the future studies.
